# Validation of the Memorial Sloan-Kettering Cancer Center Nomogram to Predict Disease-Specific Survival after R0 Resection in a Chinese Gastric Cancer Population

**DOI:** 10.1371/journal.pone.0076041

**Published:** 2013-10-17

**Authors:** Donglai Chen, Beihai Jiang, Jiadi Xing, Maoxing Liu, Ming Cui, Yiqiang Liu, Zaozao Wang, Lei Chen, Hong Yang, Chenghai Zhang, Zhendan Yao, Nan Zhang, Jiafu Ji, Hong Qu, Xiangqian Su

**Affiliations:** 1 Center for Bioinformatics, State Key Laboratory of Protein and Plant Gene Research, College of Life Sciences, Peking University, Beijing, China; 2 Key laboratory of Carcinogenesis and Translational Research (Ministry of Education), Department of Minimally Invasive Gastrointestinal Surgery, Peking University Cancer Hospital & Institute, Beijing, China; 3 Key laboratory of Carcinogenesis and Translational Research (Ministry of Education), Department of Pathology, Peking University Cancer Hospital & Institute, Beijing, China; 4 Key laboratory of Carcinogenesis and Translational Research (Ministry of Education), Department of Surgery, Peking University Cancer Hospital & Institute, Beijing, China; Yonsei University College of Medicine, Korea, Republic of

## Abstract

**Background:**

Prediction of disease-specific survival (DSS) for individual patient with gastric cancer after R0 resection remains a clinical concern. Since the clinicopathologic characteristics of gastric cancer vary widely between China and western countries, this study is to evaluate a nomogram from Memorial Sloan-Kettering Cancer Center (MSKCC) for predicting the probability of DSS in patients with gastric cancer from a Chinese cohort.

**Methods:**

From 1998 to 2007, clinical data of 979 patients with gastric cancer who underwent R0 resection were retrospectively collected from Peking University Cancer Hospital & Institute and used for external validation. The performance of the MSKCC nomogram in our population was assessed using concordance index (C-index) and calibration plot.

**Results:**

The C-index for the MSKCC predictive nomogram was 0.74 in the Chinese cohort, compared with 0.69 for American Joint Committee on Cancer (AJCC) staging system (*P*<0.0001). This suggests that the discriminating value of MSKCC nomogram is superior to AJCC staging system for prognostic prediction in the Chinese population. Calibration plots showed that the actual survival of Chinese patients corresponded closely to the MSKCC nonogram-predicted survival probabilities. Moreover, MSKCC nomogram predictions demonstrated the heterogeneity of survival in stage IIA/IIB/IIIA/IIIB disease of the Chinese patients.

**Conclusion:**

In this study, we externally validated MSKCC nomogram for predicting the probability of 5- and 9-year DSS after R0 resection for gastric cancer in a Chinese population. The MSKCC nomogram performed well with good discrimination and calibration. The MSKCC nomogram improved individualized predictions of survival, and may assist Chinese clinicians and patients in individual follow-up scheduling, and decision making with regard to various treatment options.

## Introduction

Although gastric cancer rates have decreased substantially over the past few decades, the disease remains one of the most frequent malignancies worldwide. Over 70% of new cases and deaths occur in Eastern Asia, Eastern Europe, and South America, including nearly 42% in China [1], [2]. Surgery remains the primary curative treatment for gastric cancer without metastasis [3], [4]. With the addition of pre- and postoperative adjuvant therapy, 5-year survival rate of gastric cancer has been improved up to 30–35% [5], [6]. In most institutes, gastric cancer are treated based on the American Joint Committee on Cancer (AJCC) staging groups, including the clinical parameters of pathologic depth of invasion (T), the number of metastatic lymph nodes (N), and distant metastasis (M) [7]. However, because the patients within the same stage may have different prognosis, it is critical to identify the patients with high risk of relapse and poor outcome. Accordingly, the individualized therapies can be administrated for those patients.

A number of recent studies suggest that the use of the nomogram in predicting the prognosis of malignancies improved decision making in cancer therapy [8]–[12]. The nomogram incorporates multiple clinical parameters into cancer prediction models, permitting a more individualized prediction of outcome, and providing a more accurate prediction than conventional staging or scoring systems [8]–[12]. The nomograms for several types of cancer are available (http://www.mskcc.org/cancer-care/prediction-tools). For gastric cancer, although there are several nomograms for predicting individual survival, the nomogram derived from Memorial Sloan-Kettering Cancer Center (MSKCC) is the only one available online [13]–[17]. In 2003, Kattan et al. developed a nomogram for predicting the probability of disease-specific survival (DSS) after R0 resection on the basis of 1039 patients who underwent treatment at MSKCC [13]. This nomogram has been validated as an accurate prediction tool for DSS in the United States, the Netherland, and Germany [18]–[20].

However, several clinicopathologic characteristics of gastric cancer in eastern countries differ from those in western countries, which include racial diversity, environmental exposures, tumor location, surgical treatment, and adjuvant therapy patients received [21], [22]. These differences may cause inaccurate prediction of clinical outcome of eastern patients using the same nomogram model. For instance, patients from Korea appear to have consistently better DSS, compared with those from the United States when analyzed with the validated nomogram from MSKCC [21]. In addition, MSKCC nomogram was not accurate in predicting the clinical outcome of gastric cancer patients in the Turkish population [23]. Meanwhile, whether the existing MSKCC nomogram for DSS prediction is suitable for analyzing Chinese patients with gastric cancer is largely unknown. Therefore, we evaluated the performance of the nomogram by using a large and independent patient population from Peking University Cancer Hospital & Institute.

In this study, we externally validated the MSKCC nomogram predicting the probability of 5- and 9-year DSS after R0 resection for gastric cancer in a Chinese patient population. Our results suggest that the MSKCC nomogram performed well with good discrimination and calibration in predicting DSS probability of Chinese patients with gastric cancer. The prediction of the individual patient prognosis by the MSKCC nomogram serves as a reliable strategy to better counsel patients, tailor adjuvant treatment, and schedule follow-up for Chinese patients with gastric cancer.

## Materials and Methods

### Ethics Statement

The retrospective study was approved and supervised by the research ethics committee of Peking University Cancer Hospital & Institute (Beijing, China). Written Informed Consents were obtained from all patients prior to being registered in this study.

### Patients

Between June 1, 1998 and March 30, 2007, 1065 consecutive patients with gastric cancer who underwent R0 resection at Peking University Cancer Hospital & Institute were retrospectively reviewed. Neither neoadjuvant chemotherapy nor radiation therapy was performed prior to surgery for the patients during that period of time. The extent of resection for gastric cancer was total or subtotal gastrectomy with D2 lymphadenectomy according to the Japanese Research Society for Gastric Cancer (JRSGC). A R0 resection was defined as complete resection without microscopic residual tumor. Patients were excluded from the study if final pathology revealed a positive surgical margin or metastatic disease.

### Clinicopathologic Variables

Patients' data were collected including the following prognostic variables: sex, age of diagnosis, primary location (antrum or pyloric, middle one third, gastroesophageal junction, and proximal one third), Lauren histotype (diffuse, intestinal, mixed), tumor size, number of positive lymph nodes resected, number of negative lymph nodes resected, and depth of tumor invasion (mucosa, submucosa, propria muscularis, subserosa, suspected serosal invasion, definite serosal invasion, and adjacent organ involvement). Of the 1065 patients from our database, 979 patients with complete records were included in the present study, and the rest patients were excluded due to lack of information, death from cause unrelated to gastric cancer, or loss to follow-up.

### Follow-up

Patients with stage II or III gastric cancer routinely received postoperative adjuvant chemotherapy with a relatively consistent protocol based on fluorouracil and platinum in our hospital & institute. The regular follow-up program started after patients being discharged from the hospital. Patients were followed-up every 3–6 months during the first 2 years, every 6 months until the fifth postoperative year, then once every year thereafter. Follow-up evaluation consisted of physical examination, radiological studies, endoscopic examination, and laboratory examination.

### Statistical analysis

DSS was defined as the time from primary surgery to death from gastric cancer or last follow-up, and was estimated using the Kaplan-Meier method. Univariate and multivariate analysis was performed using the Cox proportional hazards regression model. The 5- and 9-year predicted probability of DSS was calculated for each patient using the MSKCC nomogram application (http://nomograms.mskcc.org/Gastric/ROResection.aspx).

The MSKCC nomogram was validated with the concordance index (C-index) and a calibration plot using the dataset from Peking University Cancer Hospital & Institute. C-index is a probability of concordance between predicted and observed survival, similar to the area under the receiver operating characteristic curve for censored data. C-index can range from 0.5 to 1.0, indicating a random predictions and perfect concordance, respectively. A Z test was used to evaluate the difference between nomogram and staging system concordance indices. A calibration plot was applied to assess the prediction accuracy of the nomogram by plotting the actual survival against the nomogram-predicted survival probabilities. All statistical analyses were performed by using the R software with rms package version 3.6-3 (http://CRAN.R-project.org/package=rms). A two-sided *P* value of less than 0.05 was considered statistically significant.

## Results

### Clinicopathologic characteristics

**Figure 1:**
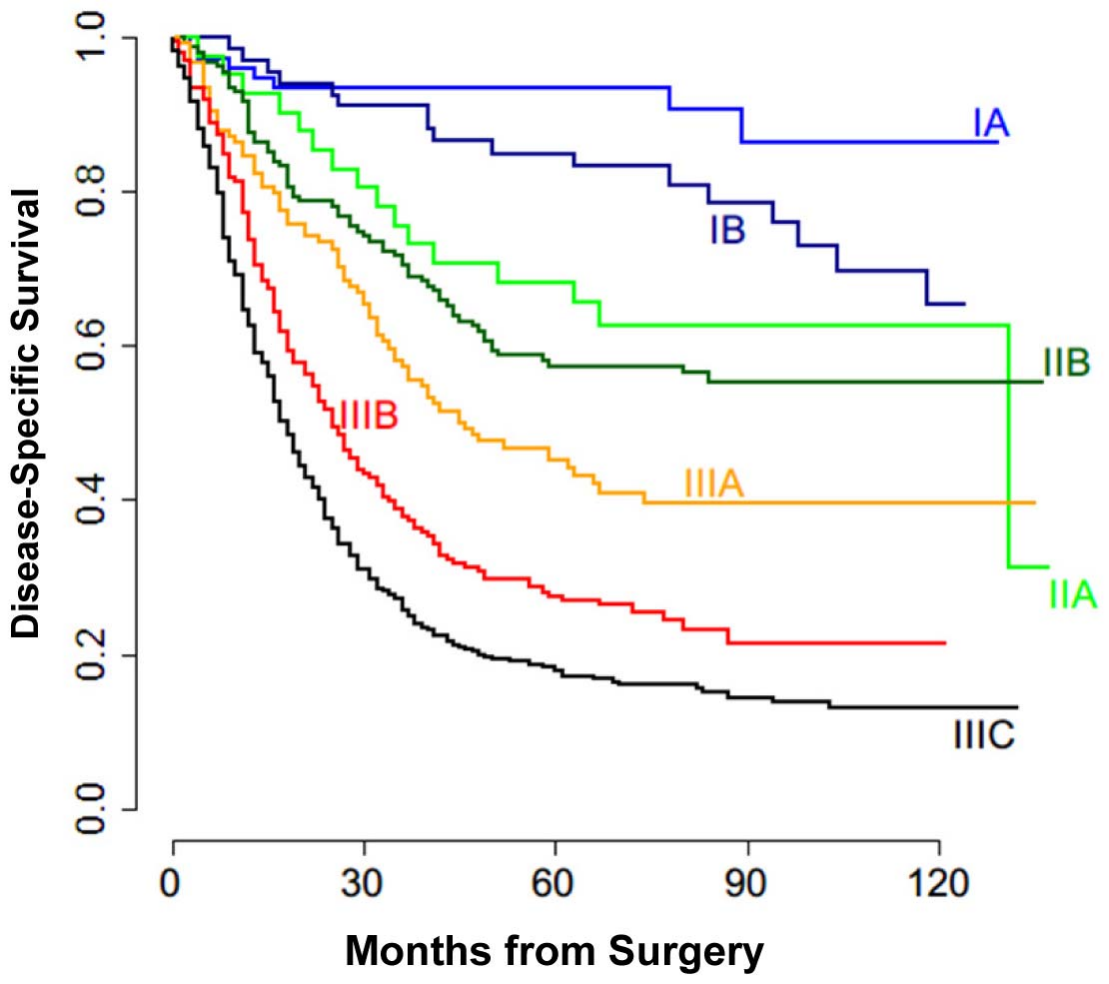
Kaplan-Meier curves of DSS according to the seventh AJCC TNM classification. Kaplan-Meier DSS curves for all patients received R0 gastric cancer resection at Peking University Cancer Hospital & Institute from 1998 to 2007. Each line represents the survival of patients within a single TNM stage.

The clinicopathologic characteristics of Chinese patients were summarized in table 1. The median age at diagnosis was 61 years (range, 20 to 87). The percentage of male (74.0%) was higher than that of female (26.0%) in the study cohort. The last follow-up date is July 17, 2012. The median follow-up period was 48 months (range, 0 to 137 months). Six hundred and two patients died of gastric cancer during this period of time. As shown in Figure 1, DSS by the seventh AJCC TNM stage grouping suggested a sufficient number of patients at risk at both 5 and 9 years to evaluate the MSKCC nomogram.

**Table 1:** Patient demographic and clinicopathologic characteristics for gastric cancer cohort.

Variables	Cases	(%)
Sex		
Male	724	74.0
Female	255	26.0
Primary location		
Antrum/Pyloric	428	43.7
Middle Third	192	19.6
GE Junction	254	25.9
Proximal Third	105	10.7
Lauren histotype		
Diffuse	445	45.5
Intestinal	355	36.3
Mixed	179	18.3
Stage		
IA	75	7.7
IB	67	6.8
IIA	41	4.2
IIB	155	15.8
IIIA	124	12.7
IIIB	197	20.1
IIIC	320	32.7
Depth of tumor invasion		
Mucosa	39	4.0
Submucosa	56	5.7
Propria Muscularis	121	12.4
Subserosa	22	2.2
Susp Serosal Invasion	207	21.1
Def. Serosal Invasion	468	47.8
Adjacent Organ Invasion	66	6.7
Number of positive nodes		
Minimum	0	
1st quartile	0	
Median	3	
Mean	6	
3rd quartile	9	
Maximum	58	
Number of negative nodes		
Minimum	0	
1st quartile	6	
Median	12	
Mean	14	
3rd quartile	19	
Maximum	74	
Size (cm)		
Minimum	0.1	
1st quartile	3.0	
Median	4.0	
Mean	5.0	
3rd quartile	6.0	
Maximum	18.5	
Age		
Minimum	20	
1st quartile	52	
Median	61	
Mean	59	
3rd quartile	68	
Maximum	87	

### Univariate and Multivariate analyses to identify DSS-associated variables

In our cohort, univariate analysis revealed that age at diagnosis, primary tumor location, Lauren histotype, tumor size, number of positive nodes retrieved, number of negative nodes retrieved, and depth of tumor invasion were significantly associated with DSS, whereas sex was not (Table 2). Multivariate analysis demonstrated that age of 61–68, tumor at GE junction, large tumor size, high number of positive nodes retrieved, low number of negative nodes retrieved, and deep tumor invasion were significantly associated with poor DSS. However, no significant correlation with DSS was found in sex or Lauren histotype (Table 2).

**Table 2:** Univariate and multivariate analysis of clinicopathologic variable influence on DSS in Chinese cohort.

Variables	Univariate			Multivariate		
	HR	95%CI	*P*	HR	95%CI	*P*
Sex						
Female	1			1		
Male	0.961	0.801–1.154	0.673	0.962	0.796–1.162	0.685
Age$						
<52	1			1		
52–61	0.963	0.757–1.224	0.756	0.930	0.728–1.189	0.564
61–68	1.282	1.017–1.616	0.035	1.274	1.003–1.618	0.047
>68	1.355	1.085–1.693	0.007	1.229	0.976–1.547	0.079
Primary location						
Antrum/Pyloric	1			1		
Middle Third	1.138	0.905–1.430	0.268	0.998	0.790–1.261	0.989
GE Junction	1.777	1.466–2.154	<0.0001	1.284	1.040–1.585	0.020
Proximal Third	1.472	1.128–1.920	0.004	0.973	0.736–1.286	0.846
Lauren histotype						
Diffuse	1			1		
Intestinal	0.786	0.655–0.943	0.009	0.846	0.698–1.025	0.088
Mixed	1.092	0.883–1.351	0.416	1.022	0.822–1.271	0.842
Size$						
<3	1			1		
3–4	1.435	1.075–1.917	0.014	0.969	0.718–1.309	0.839
4–6	1.829	1.426–2.347	<0.0001	1.066	0.817–1.392	0.637
>6	3.041	2.395–3.861	<0.0001	1.489	1.144–1.938	0.003
Number of positive nodes$						
0	1			1		
0–3	2.237	1.652–3.029	<0.0001	1.747	1.276–2.392	<0.0001
3–9	3.824	2.969–4.925	<0.0001	2.654	2.025–3.480	<0.0001
>9	6.427	4.988–8.282	<0.0001	3.670	2.773–4.858	<0.0001
Number of negative nodes$						
<6	1			1		
6–12	0.548	0.446–0.672	<0.0001	0.628	0.509–0.775	<0.0001
12–19	0.371	0.296–0.464	<0.0001	0.508	0.403–0.642	<0.0001
>19	0.193	0.148–0.250	<0.0001	0.309	0.234–0.408	<0.0001
Depth of tumor invasion						
Mucosa	1			1		
Submucosa	1.639	0.623–4.311	0.317	1.304	0.492–3.460	0.593
Propria Muscularis	2.556	1.089–5.998	0.031	1.524	0.637–3.641	0.344
Subserosa	4.865	1.849–12.802	0.001	3.253	1.215–8.708	0.019
Susp Serosal Invasion	7.152	3.161–16.180	<0.0001	2.747	1.175–6.419	0.020
Def. Serosal Invasion	7.309	3.258–16.395	<0.0001	2.639	1.136–6.129	0.024
Adjacent Organ Invasion	9.434	4.048–21.983	<0.0001	3.467	1.438–8.355	0.006

$ The values are, respectively, the 1st, 2nd, 3rd, and 4th quartiles of the variable distribution.

### Evaluation of the MSKCC nomogram

**Figure 2:**
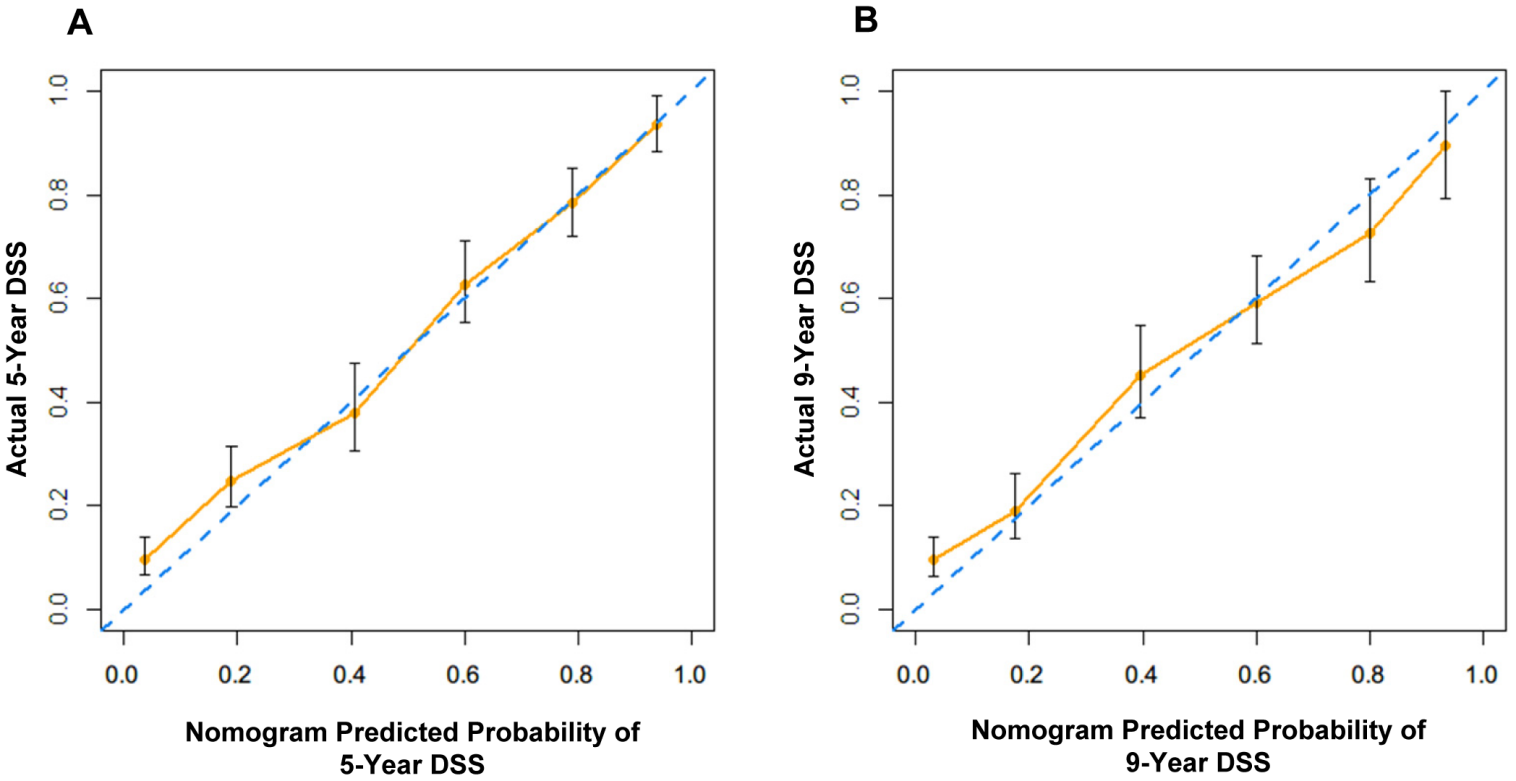
Calibration plots for the MSKCC nomogram in Chinese cohort. On the calibration plot, x-axis is the nomogram-predicted probability of DSS, and y-axis is the actual probability of DSS estimated by the Kaplan-Meier method. The dotted line represents the ideal nomogram; the solid line represents the performance of the MSKCC nomogram in Chinese cohort. (A) Five-year nomogram, (B) Nine-year nomogram.

The performance of the MSKCC nomogram in our cohort was evaluated by two methods. Firstly, the C-index was assessed to quantify the discrimination among individual patients in the MSKCC nomogram. The C-index of this model was 0.74. Secondly, the calibration plots compared the nomogram-predicted probabilities of DSS with the observed rate of DSS at 5 and 9 years (Figure 2). The performance of the ideal nomogram was plotted by the dotted line, in which the predicted outcome would perfectly overlap with the actual outcome. The solid line represents the performance of the MSKCC nomogram in predicting DSS probability of Chinese patients with gastric cancer after R0 resection. The calibration plots showed that the actual survival corresponded closely to the MSKCC nonogram-predicted survival probabilities. Although the MSKCC nomogram slightly under- or overestimated the probability of DSS in patients who had a low or high probability of the actual DSS, respectively, it was within a 10% margin of the nomogram prediction (Figure 2).

**Figure 3:**
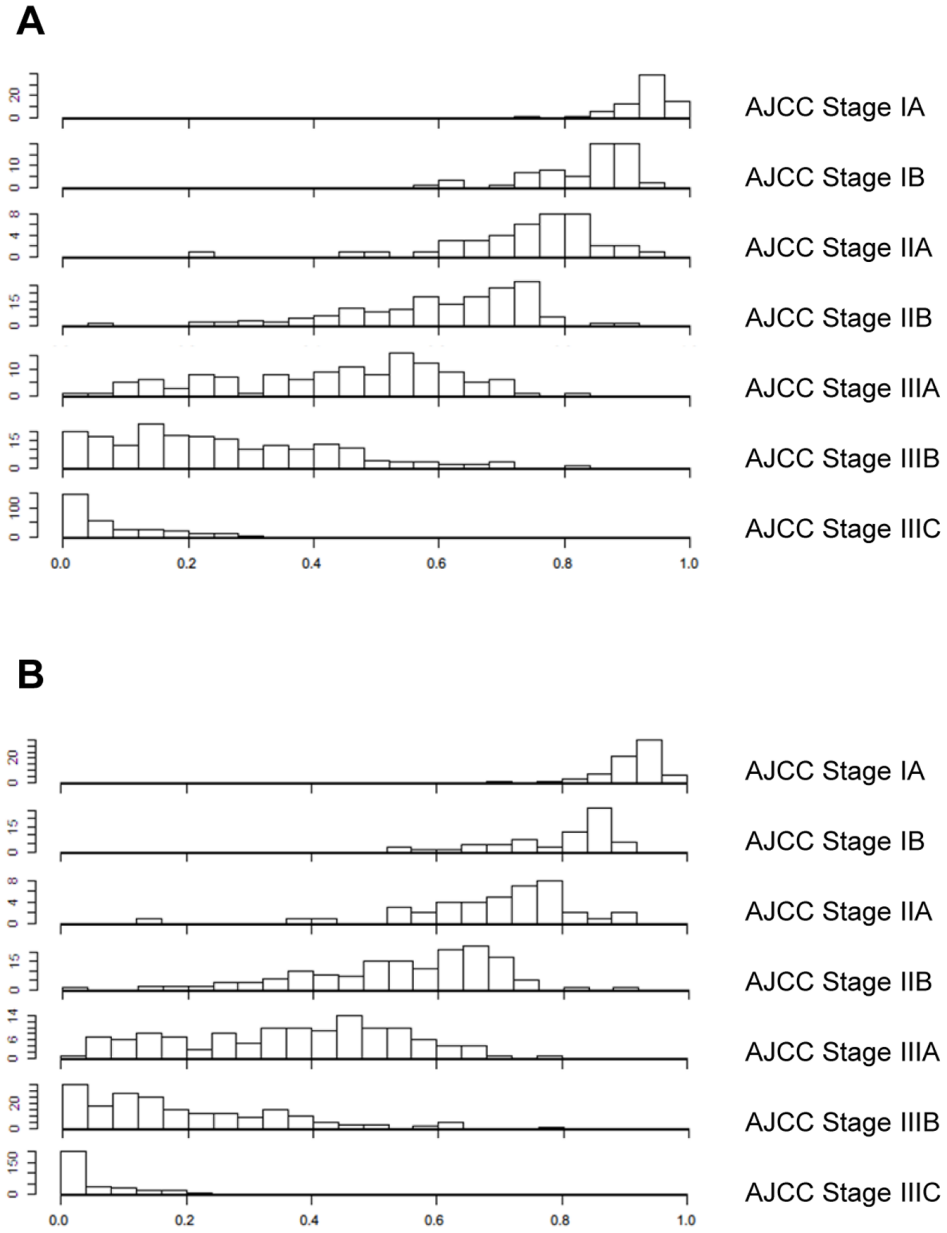
Histogram of the MSKCC nomogram-predicted probabilities within each AJCC stage. The x-axis represents AJCC stages, and the y-axis represents nomogram-calculated probability of DSS. Note the heterogeneity of predicted probabilities of survival within each AJCC stage, especially in stages IIA, IIB, IIIA, and IIIB. (A) Five-year predictons, (B) Nine-year predictions.

Then, we compared the predictive ability of MSKCC nomogram with that of the seventh AJCC stage risk grouping. The MSKCC nomogram was found to be quantitatively more discriminative than the seventh AJCC TNM classification (C-index 0.74 versus 0.69; *P*<0.0001). To illustrate the discrepancies between the two predicting methods, Figure 3 showed a histogram of nomogram-predicted survival probabilities for each AJCC stage, suggesting the heterogeneity within several of the AJCC stages, particularly stages IIA, IIB, IIIA, and IIIB. When the histograms of the nomogram predicted probabilities are compared, every AJCC stage overlaps with the neighboring AJCC stages. This suggests that patients of stages IIA, IIB, IIIA, and IIIB have far more variable survival than expected according to AJCC stage alone.

**Figure 4:**
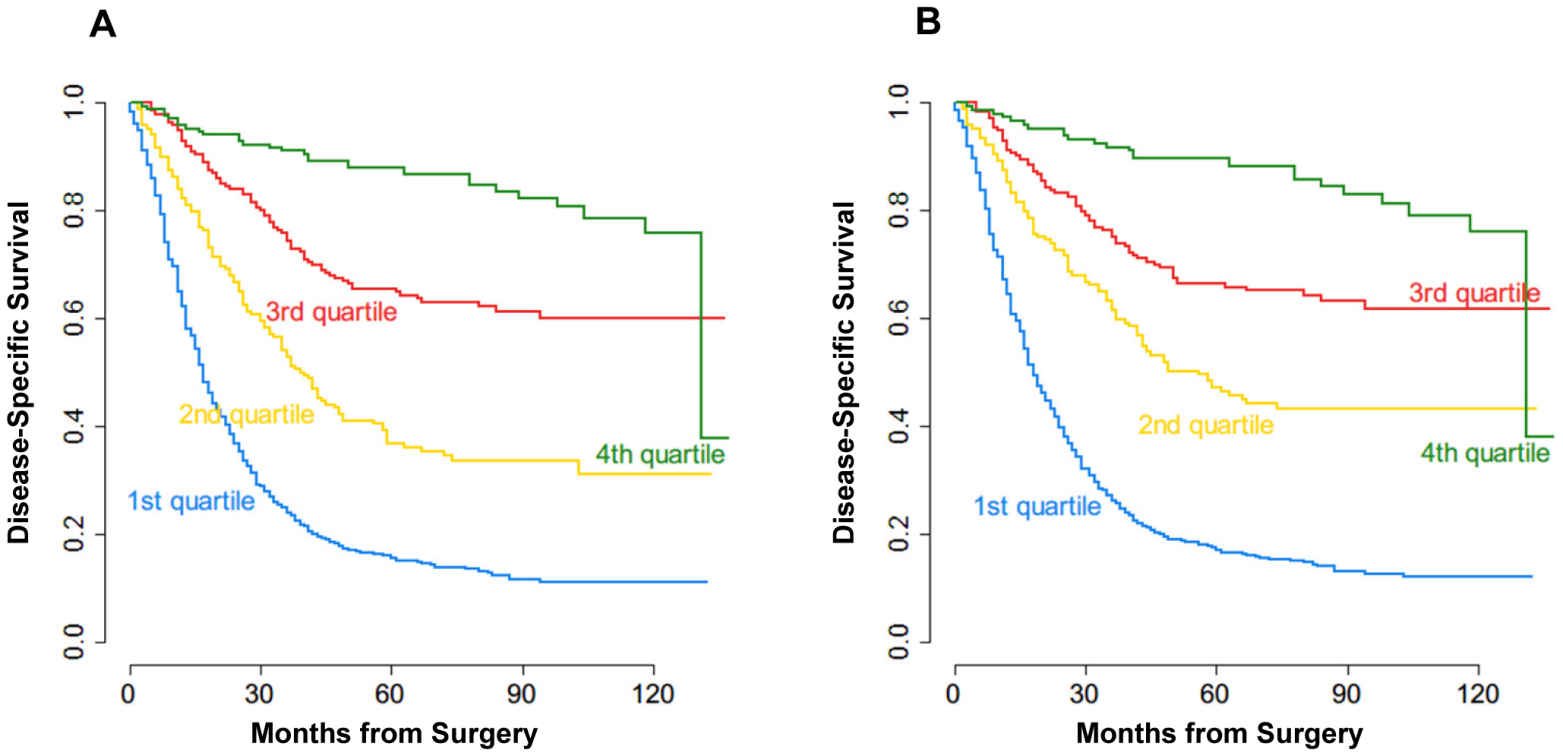
Kaplan-Meier curves for all patients stratified by quartiles of the MSKCC nomogram-predicted survival. Patients in the Chinese cohort were grouped into quartiles according to the nomogram-predicted survival (1st quartile, <25%; 2nd quartile, 25–50%; 3rd quartile, 50–75%; 4th quartile, >75%). (A) Five-year survival (B) Nine-year survival.

To further evaluate the MSKCC nomogram in our cohort, patients were grouped into quartiles according to the nomogram-predicted survival (1st quartile, <25%; 2nd quartile, 25–50%; 3rd quartile, 50–75%; 4th quartile, >75%). Figure 4 showed the observed survival curves for the four risk groups. The quartiles of nomogram-predicted survival were significantly associated with different observed survival probabilities (*P*<0.0001, *P*<0.0001, for 5 years, and 9 years, respectively, Figure 4).

## Discussion

Gastric cancer is the second major cause of cancer-related deaths worldwide. In China, an estimated 380,000 new cases each year account for more than 40% of the global annual gastric cancer incidence [1], [2], [24]. For patients who underwent R0 resection, AJCC stage is the commonly used system to predict prognosis [7]. However, other factors like sex, age of diagnosis, primary tumor location, Lauren histotype, and tumor size, which may have impacts on DSS, were not taken into account in the AJCC stage grouping. A nomogram, which combined all proven prognostic factors and quantified risks, was developed by Kattan et al, and has been validated to be more accurate for predicting DSS rate of gastric cancer in the United States, the Netherland, and Germany [13], [18]–[20], whereas this nomogram has not been evaluated in Chinese patients. Thus, we determined the predictive value of the MSKCC nomogram for Chinese patients with gastric cancer. Our analysis showed that the predictive value of the MSKCC nomogram remained accurate in a Chinese population of patients with gastric cancer.

In the current study, the validation of the MSKCC nomogram was performed by using C-index and calibration plots. The results showed that the MSKCC nomogram predicts the probability of DSS in gastric cancer patients with a C-index of 0.74, which is more accurate than the seventh AJCC staging system (C-index, 0.69). The results suggested that the discrimination of patients by nomogram is superior to that of AJCC stage grouping (*P*<0.0001). In addition, the calibration plots of the MSKCC nomogram for the Chinese cohort appeared to be accurate for both 5- and 9-year prediction.

The Chinese and MSKCC patients were comparable in the distribution of all clinicopathologic variables. There are several differences in clinicopathologic characteristics between Chinese and MSKCC cohort (Table S1). Firstly, a significant majority of patients in the Chinese cohort were male, compared with female in the MSKCC cohort. Secondly, primary tumor location is commonly seen at antrum or pyloric in the Chinese cohort, compared with gastroesophageal junction (GEJ), which indicates poor survival, in the MSKCC cohort. Thirdly, the Lauren histotype is different, with high percentage of diffuse type in the Chinese cohort, compared with intestine type in the MSKCC cohort. In addition, although the Chinese cohort and the MSKCC cohort used the 7th and 5th AJCC TNM classification, respectively, Chinese patients have the tendency to be diagnosed with more advanced gastric cancer with higher T stage (depth of tumor invasion), and more positive lymph nodes retrieval for R0 resection, compared with the MSKCC cohort in Kattan's study [13]. Moreover, when comparing treatment, Chinese patients with stage II or III tumor routinely received postoperative adjuvant chemotherapy, the patients from MSKCC cohort underwent no adjuvant treatment [13], [19]. Despite these discrepancies between the two cohorts, the MSKCC nomogram was validated as a more accurate survival predictor than AJCC staging system for Chinese patients in our institute.

The improved accuracy of the nomogram prediction is due to the fact that it incorporates many DSS-related variables that are not considered in the AJCC classification. In the study by Kattan et al, sex, age of diagnosis, primary tumor location, Lauren histotype, and tumor size were included as prognostic factors for the development of MSKCC nomogram [13]. Moreover, for some patients, the changes in prognostic predictions suggest good clinical utility. Regarding the AJCC TNM classification, patients within the same stage would be assigned for the same prognosis. However, nomogram predictions separated patients within the same stage into different survival prognosis, and allowed better discrimination among patients. Accurate survival predictions by nomogram may assist in individual patient counseling and in follow-up scheduling. It may also play roles in identifying patients with high risk of poor clinical outcome within known AJCC stages, as well as in selecting patients who may benefit from certain adjuvant treatments.

However, there are limitations of this study. The MSKCC nomogram was externally validated using a retrospective data set from a single Chinese institute. For the generalized use of the MSKCC nomogram for Chinese patients, validation by other Chinese cohorts is required. Moreover, the application of the nomogram depends on several pathologic variables that only available after surgery, i.e. Lauren histotype, depth of tumor invasion and number of positive or nodes retrieved. Thus, it is difficult to make a precise assessment of these factors preoperatively. Therefore, the nomogram has limited impact with regard to alternative treatments before surgery, including the use of neoadjuvant chemotherapy.

In conclusion, on the basis of a single Chinese cohort database, we externally validated the MSKCC nomogram in predicting the probability of 5- and 9-year DSS after R0 resection for gastric cancer. The MSKCC nomogram performed well with a good discrimination and calibration, which makes it a simple and easy tool to set up an individualized estimation of survival for Chinese patients with gastric cancer. The MSKCC model could be useful for clinicians in terms of better counseling patients, tailoring adjuvant treatment, as well as scheduling follow-up.

## Supporting Information

Table S1 **Comparison of patient clinicopathologic characteristics in the Chinese and MSKCC cohort.**
(DOC)
