# Supplementary material for: Validation of the Memorial Sloan-Kettering Cancer Center Nomogram to Predict Disease-Specific Survival after R0 Resection in a Chinese Gastric Cancer Population
Source: PLoS One. 2013 Oct 17;8(10):e76041. doi: 10.1371/journal.pone.0076041 (PMC3798309; doi:10.1371/journal.pone.0076041)
Supplement: Table S1 — Comparison of patient clinicopathologic characteristics in the Chinese and MSKCC cohort. (DOC) [file pone.0076041.s001.doc]

**Supporting Information**

**Table S1.** Comparison of patient clinicopathologic characteristics in the Chinese and MSKCC cohort.

| Variables | Chinese cohort (%) | MSKCC cohort (%) ^$^ | *P* value^&^ |
| --- | --- | --- | --- |
| Gender |  |  |  |
| Male | 724(74.0) | 415(36.5) | <0.0001 |
| Female | 255(26.0) | 721(63.5) |  |
| Primary location |  |  |  |
| Antrum/Pyloric | 428(43.7) | 325(28.6) | <0.0001 |
| Middle Third | 192(19.6) | 248(21.8) |  |
| GE Junction | 254(25.9) | 376(33) |  |
| Proximal Third | 105(10.7) | 187(16.5) |  |
| Lauren histotype |  |  |  |
| Diffuse | 445(45.5) | 359(31.6) | <0.0001 |
| Intestinal | 355(36.3) | 641(56.4) |  |
| Mixed | 179(18.3) | 98(8.6) |  |
| Not available |  | 38(3.3) |  |
| Depth of tumor invasion |  |  |  |
| Mucosa | 39(4.0) | 94(8.3) | <0.0001 |
| Submucosa | 56(5.7) | 156(13.7) |  |
| Propria Muscularis | 121(12.4) | 138(12.1) |  |
| Subserosa | 22(2.2) | 245(21.6) |  |
| Susp Serosal Invasion | 207(21.1) | 24(2.1) |  |
| Def. Serosal Invasion | 468(47.8) | 389(34.2) |  |
| Adjacent Organ Invasion | 66(6.7) | 40(3.5) |  |
| Number of positive nodes |  |  |  |
| Minimum | 0 | 0 |  |
| 1st quartile | 0 | 0 |  |
| Median | 3 | 1 |  |
| Mean | 6 | 4 |  |
| 3rd quartile | 9 | 6 |  |
| Maximum | 58 | 60 |  |
| Number of negative nodes |  |  |  |
| Minimum | 0 | 0 |  |
| 1st quartile | 6 | 10 |  |
| Median | 12 | 17 |  |
| Mean | 14 | 20 |  |
| 3rd quartile | 19 | 27 |  |
| Maximum | 74 | 84 |  |
| Not available |  | 3 |  |
| Size |  |  |  |
| Minimum | 0.1 | 0 |  |
| 1st quartile | 3.0 | 2.5 |  |
| Median | 4.0 | 4.3 |  |
| Mean | 5.0 | 4.8 |  |
| 3rd quartile | 6.0 | 6.5 |  |
| Maximum | 18.5 | 21 |  |
| Not available |  | 27 |  |

$ These data were cited from Kattan’s article [13].

& *P* values were obtained using the chi-square test.
